# Diabetes Mellitus Versus Hypertension—Does Disease Affect Pharmacological Adherence?

**DOI:** 10.3389/fphar.2020.01157

**Published:** 2020-08-10

**Authors:** Beata Jankowska-Polańska, Piotr Karniej, Jacek Polański, Mariola Seń, Natalia Świątoniowska-Lonc, Elżbieta Grochans

**Affiliations:** ^1^Department of Clinical Nursing, Public Health Department, Wroclaw Medical University, Wrocław, Poland; ^2^Department of Health Promotion, Public Health Department, Wroclaw Medical University, Wrocław, Poland; ^3^Department of Internal Medicine, Occupational Diseases, Hypertension and Clinical Oncology, Wroclaw Medical University, Wrocław, Poland; ^4^Department of Nursing, Pomeranian Medical University in Szczecin, Szczecin, Poland

**Keywords:** adherence, hypertensive treatment, hypertension, diabetes, factors

## Abstract

The aim of the study was to answer the questions whether a chronic disease can have a significant impact on the level of adherence and whether there are differences in adherence-related predictors depending on the chronic disease. The study included 1,571 patients (mean age 64.7 ± 11.3) with chronic diseases [1,030 diabetes mellitus (DM) type 2 and 541 hypertension (HA)]. Adherence was assessed using the Adherence Refills Medication Scale (ARMS). The average adherence score for the whole group was 18.9. Fifty-five percent of patients had a low level of adherence. A comparison between DM and HA shows a statistically significant difference and a higher level of adherence with pharmacological recommendations in the group of patients with type 2 DM (17.5 ± 12.0 vs 19.2 ± 8.0). In the single factors analysis, HA diagnosis had a statistically significant negative effect on adherence (β=0.92, p ≤ 0.001). In simple linear regression analysis, independent of chronic disease, a higher level of adherence was observed among women (β=−0.40, p=0.015), people with secondary education (β=−1.26, p ≤ 0.001), and inactive patients (β=−0.48; p=0.005). However, place of residence - countryside (β =0.35, p=0.044) and higher education (β=0.90, p ≤ 0.001) had a negative influence on the level of adherence. In multiple linear regression analysis HA (B=0.99; p ≤ 0.001), female gender (B=−0.47; p=0.003) and secondary education (B=−1.16; p ≤ 0.001) were important independent determinants of adherence. (1) Hypertension is an independent, statistically significant predictor that reduces the adherence level. (2) Female gender and higher education are the most important determinants improving adherence to pharmacological therapy. (3) There is a different pattern of predictors of adherence among patients: occupational activity plays an important role in DM, while education plays a role in HA.

## Introduction

Chronic diseases are the most common cause of death worldwide; they develop unnoticed over a long period and are usually diagnosed at an advanced stage of development. The treatment of chronic diseases involves a considerable proportion of the budget for general expenditure related to health care systems. The most common chronic diseases include rheumatic diseases, chronic obstructive pulmonary disease, hypertension, heart failure and diabetes (Jansà et al., 2010). Hypertension (HA) or diabetes mellitus (DM) are the most common risk factors for cardiovascular diseases, strokes, and chronic kidney disease (Centers for Disease Control and Prevention, 2011; Menke et al., 2015; American Diabetes Association, 2017). Currently, 3 million people in Poland suffer from DM and almost 10 million from HA. In 2018, due to HA, 20.4 million consultations within the framework of basic health care, 2.8 million consultations within the framework of outpatient specialist care and 877,000 hospitalizations within the framework of hospital treatment occurred (NFZ, 2018). The occurrence of HA and DM is also a major economic problem and is associated with high treatment costs. Although effective treatments are widely available, about half of the patients treated do not have adequate blood pressure (BP) or balanced metabolic control (Gupta and Guptha et al., 2010). The literature indicates that about 20–30% of drugs prescribed will never be purchased, and 50% of patients will cease their therapy prematurely within a year of starting treatment.

According to the WHO (World Health Organization), the effectiveness of adherence interventions can have a significant impact on patient self-management of chronic diseases. (WHO, 2003).

The main causes of treatment failure are a low level of pharmacological adherence, a lack of patient engagement, and therapeutic inertia. Non-adherence is extremely costly and very difficult to assess. It is even more difficult to plan corrective actions (Lloyd et al., 2019). The consequences of non-adherence in both chronic diseases have critical long-term consequences for both the patient and the national health service budget. The psychological literature points out that people are “poor intuitive statisticians”, because they are not able to estimate the risk of consequences they may suffer as a result of non-adherence and poor control of chronic diseases (Peterson et al., 2003).

In Poland, in 2017, about 26,000 patients died from complications of DM, the most common cause being deaths due to cardiovascular complications caused by DM (about 70%). The reasons for low treatment efficacy are high drug prices, lack of reimbursement, and low sales due to the poor financial state of Polish patients (Ambroziewicz et al., 2019). Adherence, taking medication on a regular basis and changing one’s lifestyle leads to a reduction in symptoms, complications, and to an improvement in quality of life, as well as significantly reducing the costs of treatment during rehabilitation (Mirkarimi et al., 2018). According to the WHO, preventing non-adherence by implementing appropriate interventions can have a much greater impact on the health of the population than the improving medical therapy (WHO, 2003).

Understanding and improving adherence in the treatment of chronic diseases is essential to controlling and achieving clinical alignment. A high level of adherence significantly reduces the number of complications. Non-adherence leads to increased mortality (Ho et al., 2006). Nonetheless, the problem of adherence remains unresolved. There is a discussion in the literature on the factors that affect the level of pharmacological adherence. The most frequently mentioned factors include beliefs related to the treatment used, lack of visible benefits from the treatment used, side effects and adverse reactions, and difficulties in understanding and adapting to the recommendations that are set. Furthermore, attention should be paid to the cost of treatment and the confusion about the routes and times of administration (Holman et al., 2008; Mayberry et al., 2013).

Many researchers are looking for factors related to adherence with therapeutic recommendations, especially pharmacological ones, and opportunities for improvement. However, the results presented are contradictory and there is a discussion in the literature about the relationship between sex, age, professional activity, multi-medication, and concomitant diseases. There is a lack of information regarding an answer to the question of whether the type of chronic disease affects the level of adherence and whether the profile of adherence-related factors in each chronic disease is the same. Can we generalize and assume that the same problems of non-adherence should be addressed in each case? There are no studies assessing and comparing the level of adherence and determinants of adherence in particular chronic diseases. It can be assumed that the level of adherence in each chronic disease is limited, as are the predictive factors that strengthen or weaken adherence.

Primarily, the aim of the current research is in line with the ongoing discussion and attempts to understand which sociodemographic factors are related to adherence in a group of chronic patients. Secondly, it is important to answer the question of whether a chronic disease such as HA or DM can have a significant impact on the level of adherence and whether there are differences in adherence-related predictors depending on the chronic disease.

## Methods

### Design and Setting

The present research has a cross-sectional, observational, and multi-center study design. Two institutions were involved: Department of Clinical Nursing of Medical University of Wroclaw (Poland) and Faculty of Health Sciences of Pomeranian Medical University (Poland). Data were collected from January 2019 to December 2019. The study used a closed-ended standardized survey and 1-on-1 interviews.

### Participants

The study involved 1,571 inpatients who had been diagnosed with HA in accordance with the European Society of Hypertension guidelines (Williams et al., 2018) and/or type 2 DM, according to the Polish Diabetes Association (Araszkiewicz et al., 2019).

### Intervention

All patients had been treated with at least one drug for 6 months or more, were over 18 years of age, and had no mental disorders or cognitive impairment with dementia or no concomitant chronic disease (Cumulative Illness Rating Scale< 1) or a severe medical condition (i.e. chronic heart failure—New York Heart Association IV, ischemic heart disease Canadian Cardiovascular Society IV, neoplastic disease, acute respiratory disease) or cognitive impairment were excluded.

Participant selection was conducted by a panel consisting of internal medicine physicians and nurses, who performed at least two BP measurements, a fasting blood glucose level, and a comprehensive physical examination. The patients’ clinical history was also taken from medical data. After receiving informed about the aim and course of the study, the patients gave their written informed consent to participate in the study. Following the collection of sociodemographic information (i.e. age, gender, educational level, marital status) and clinical data (i.e. BP, the presence of specific disease conditions, the total number of medical prescriptions, the duration of the main disease other medical conditions), the patients were divided into two groups according to the following: the underlying disease (type 2 DM or HA), gender (women, men), marital status (single, married), education (primary school: 6 years of primary school and 3 years of junior high school; secondary: 2–5 years, high school, technical school, vocational school; higher: 3–5 years at university), employment status (out of work, in work), place of residence (city, countryside), and age (classification according to the WHO definition: <65 and ≥65 years old) (WHO, 2001). The results of BP and fasting blood glucose level were compared with the standards proposed by the guidelines for fasting glycemia 80–110 mg/dl, and for the BP <140/90 (Williams et al., 2018; Araszkiewicz et al., 2019). During the examination, two BP measurements were performed and the mean value was calculated. During a regular follow-up visit, patients responded independently to questions referring to information from the last 4 weeks.

The ARMS produced by Kripalani et al. has been tested in patients with coronary artery disease, HA, dyslipidemia, and DM. The questionnaire consists of 12 statements consisting of two subscales: adherence to drug recommendations and “adherence to refilling prescription”. Each question can be answered by the patient “(1) never”, “(2) rarely”, “(3) often”, (4) “most of the time”. The answers to the questions are shown on the Likert scale. In order to obtain an overall assessment of adherence, the points from all 12 questions should be added together. Patients can score 12–48 points. The higher the number of points, the better the level of adherence (Kripalani et al., 2009).

Following the assessment of the cognitive functioning aimed to identify the eligible patients, self-report questionnaires evaluating pharmacological and non-pharmacological adherence and the psycho-social characteristics of the patients to be considered as possible determinants of non-adherence were administered by nurses.

### Statistical Methods

The statistical analysis was performed with Statistica 13 software (TIBCO, USA). Arithmetic means, standard deviations, and a range of variability (extreme values) were calculated for measurable variables. The frequency of occurrence (percentage) was calculated for the quality variables. All quantitative type variables were tested with the Shapiro-Wilk test to determine the type of distribution. The comparison of qualitative variables between groups (HA vs. DM type 2) was made using the chi-squared test (χ^2^). The comparison of quantitative type variables between the HA group and DM group was made using the independent-samples Student’s t-test. Comparison of ARMS results depending on selected factors were statistically analyzed by two-way ANOVA in both HA vs. DM groups.

Additionally, the influence of selected factors on adherence measured with the ARMS questionnaire and using linear regression (the model of single-factor predictors included in the analysis) was analyzed. The levels of non-standardized and standardized regression factor, standard error, and statistical significance were determined. The next step was to build a multi-factor model (progressive step method), taking into account the following variables: disease, gender, age, place of residence, marital status, education, and employment status.

### Ethical Considerations

The study was approved by the local Bioethics Committee (Approval No 730/2019). The study was performed in accordance with the Helsinki Declaration and the principles of good clinical practice, with respect for the rights and dignity of the participants. All participants provided written informed consent.

## Results

All the characteristics were prepared taking into account a cohort for patients with HA (n=1,030) and DM type 2 (n=541). A sociodemographic analysis showed that the group of patients with DM was older than the one with HA (64.2 ± 12.2 vs. 65.7 ± 9.0; p=0.009), In the group of people with HA statistically significantly more were women (p<0.001), and a significantly larger proportion of the study participants in this group had higher education (45% vs. 8%; p<0.001). Moreover, the vast majority of patients with DM remained outside the labor force (78% vs. 60% with HA; p<0.001) (**Table 1**).

**Table 1:** Sociodemographic characteristics - intergroup comparison (hypertension vs. diabetes).

		Hypertension (n=1,030)				Diabetes (n=541)				p value
		$\bar{x}$	Min	Max	SD	$\bar{x}$	Min	Max	SD	
**Age [years]**		64.2	21.0	96.0	12.2	65.7	29.0	92.0	9.0	0.009*
**Gender**	Women	n=582 (57%)				n=239 (44%)				<0.001**
	Men	n=447 (43%)				n=302 (56%)				
**Address of residence**	Countryside	n=330 (32%)				n=193 (36%)				0.15**
	City	n=700 (68%)				n=348 (64%)				
**Marital status**	Single	n=315 (31%)				n=173 (32%)				0.57**
	Married	n=715 (69%)				n=368 (68%)				
**Education**	Primary or none	n=201 (22%)				n=270 (50%)				<0.001**
	Secondary	n=362 (35%)				n=228 (42%)				
	Higher	n=466 (45%)				n=43 (8%)				
**Employment status**	Out of work	n=615 (60%)				n=421 (78%)				<0.001**
	In work	n=415 (40%)				n=120 (22%)				

n, number of people; *$\bar{x}$*, mean; Min, minimum value; Max, maximum value; SD, standard deviation; *test t; **chi^2^ test. Statistically significant results were highlighted in bold (p < 0.05).

The analysis of adherence showed that statistically significantly higher results were observed in group HA (19.2 vs. 17.5; higher score, worse adherence; p<0.001) (**Table 2**). Although there was a significant difference in the total adherence score, there was no difference between low and high adherence in the groups studied (p=0.97). The same percentage of patients with DM and HA had a low level of adherence (55% of subjects in a given group) (**Table 2**).

**Table 2:** Adherence characteristics inter-group comparison.

		Hypertension (n=1,030)		Diabetes (n=541)		p value
		$\bar{x}$	SD	$\bar{x}$	SD	
***ARMS - sum [pts]		19.2	7.1	17.5	4.6	<0.001*
**ARMS**	High adh (8-15 pts)	n=460 (45%)		n=241 (45%)		0.97**
	Low adh (16-48 pts)	n=570 (55%)		n=300 (55%)		

ARMS, the Adherence to Refilla and Medication Scale; adh, adherence; n, number of people; $\bar{x}$, mean; Min, minimum value; Max, maximum value; SD, standard deviation; *t test; ** chi^2^ test; ***The original version of the Adherence to Refills and Medications Scale is held by Sunil Kripalani, Vanderbilt University, Nashville, TN, USA. Polish version has been prepared with the consent of Kripalani S., available from Prof. Beata Jankowska-Polańska, Wroclaw Medical University, e-mail: beata.jankowska-polanska@umed.wroc.pl (Lomper et al., 2018). Statistically significant results were highlighted in bold (p < 0.05).

In the next part of the study, an analysis of adherence level was carried out depending on selected sociodemographic variables.

Women in HA and DM group had lower score of ARMS than men (**Table 3**). In addition, statistically significant differences between the groups we observed, in women and men in the HA group, the results are lower than in the DM group (**Table 4**). However, no statistically significant differences were found depending on the factor and group together (**Table 4**). Moreover, in the group with DM, statistically significant differences were observed in the number of women who achieved a high level of adherence in comparison with men with DM (52% vs. 39%) (**Tables 3** and **5**).

**Table 3:** Adherence characteristics inter-group comparison (hypertension vs. diabetes) depending on the total score of ARMS.

Factors	Group							
	Hypertension (n=1,030)				Diabetes (n=541)			
	n	$\bar{x}$	SD	p*	n	$\bar{x}$	SD	p*
**Gender**				**0.022**				**0.013**
** Women**	582	18.8	6.6		239	16.8	4.2	
** Men**	447	19.8	7.7		302	17.8	4.8	
**Place of residence**				**0.018**				0.79
** Countryside**	330	20.0	7.3		193	17.4	4.5	
** City**	700	18.8	7.0		348	17.3	4.6	
**Marital status**				0.68				0.76
** Single**	315	19.3	7.4		173	17.3	4.9	
** Married**	715	19.1	7.0		368	17.4	4.5	
**Education level**				**<0.001**				0.57
** Primary or none**	201	21.5	7.9		270	17.1	4.4	
** Secondary**	362	17.3	5.9		282	17.6	4.7	
** Higher**	466	19.7	7.3		43	17.6	4.9	
**Employment status**				0.34				**0.010**
** Out of work**	615	18.8	6.6		421	16.8	4.2	
** In work**	415	19.8	7.7		120	17.8	4.8	
**Age**				0.44				0.65
**<65 years old**	821	19.0	7.1		821	17.3	4.8	
** ≥65 years old**	749	19.4	7.1		749	17.4	4.4	

n, number of people; *$\bar{x}$*, mean; Min, minimum value; Max, maximum value; SD, standard deviation; *intra-group comparison (t-test or one-way ANOVA). Statistically significant results were highlighted in bold (p < 0.05).

**Table 4:** Results of the two-way ANOVA for comparison of groups and factors.

Factors	Factor	Group	Factor x Group
	p**	p**	p**
**Gender**	**0.003**	**<0.001**	0.94
** Women**			
** Men**			
**Place of residence**	0.08	**<0.001**	0.16
** Countryside**			
** City**			
**Marital status**	0.92	**<0.001**	0.65
** Single**			
** Married**			
**Education level**	**<0.001**	**<0.001**	**<0.001**
** Primary or none**			
** Secondary**			
** Higher**			
**Employment status**	**0.033**	**<0.001**	0.31
** Out of work**			
** In work**			
**Age**	0.44	**<0.001**	0.81
**<65 years old**			
** ≥65 years old**			

**intergroup comparison (two-way ANOVA). Statistically significant results were highlighted in bold (p < 0.05).

**Table 5:** Adherence characteristics inter-group comparison (hypertension vs. diabetes) depending on the adherence level.

Factors	Hypertension (n=1,030)			Diabetes(n=541)			p**
	High adh(8–15 pts)	Low adh(16–48 pts)	p*	High adh(8–15 pts)	Low adh(16–48 pts)	p*	
**Gender**							>0.05
** Women**	n=265 (45%)	n=317 (55%)	0.50	n=123 (52%)	n=116 (48%)	**0.004**	
** Men**	n=194 (43%)	n=253 (57%)		n=118 (39%)	n=184 (61%)		
**Place of residence**							>0.05
** Countryside**	n=130 (39%)	n=200 (61%)	**0.020**	n=80 (42%)	n=113 (59%)	0.28	
** City**	n=330 (47%)	n=370 (53%)		n=161 (46%)	n=187 (54%)		
**Marital status**							>0.05
** Single**	n=147 (47%)	n=168 (53%)	0.39	n=80 (46%)	n=93 (54%)	0.58	
** Married**	n=313 (44%)	n=402 (56%)		n=161 (44%)	n=207 (56%)		
**Education level**							>0.05
** Primary or none**	n=76 (38%)	n=125 (62%)	**0.001**	n=125 (46%)	n=145 (54%)	0.62	
** Secondary**	n=192 (41%)	n=274 (59%)		n=20 (47%)	n=23 (53%)		
** Higher**	n=192 (53%)	n=170 (47%)		n=96 (42%)	n=132 (58%)		
**Employment status**							>0.05
** Out of work**	n=285 (46%)	n=330 (54%)	0.19	n=196 (47%)	n=225 (53%)	0.08	
** In work**	n=175 (42%)	n=240 (58%)		n=45 (37%)	n=75 (63%)		
**Age**							>0.05
**<65 years old**	n=222 (45%)	n=266 (55%)	0.61	n=111 (48%)	n=130 (42%)	0.16	
** ≥65 years old**	n=238 (44%)	n=304 (56%)		n=120 (52%)	n=180 (58%)		

n, number of people; $\bar{x}$, *average; Min, minimum value; Max, maximum value; SD, standard deviation*.

*intra-group comparison (chi2 test); **intergroup comparison (chi2 test include Bonferroni correction). Statistically significant results were highlighted in bold (p < 0.05).

In a comparative analysis of the adherence level depending on the chronic disease, differences were only observed in the group of patients with HA, where a higher level of adherence was obtained by people with HA living in the city as compared to patients from the countryside (18.8 vs. 20.0) (**Table 3**).

The analysis of the level of adherence depending on marital status has shown that it is not a variable that plays a statistically significant role in adherence in this particular group of patients (**Tables 3** and **4**).

Significant differences in adherence levels depending on the chronic disease and education were observed (p<0.001; **Table 4**). Also in patients with HA, people with secondary education achieved a higher level of adherence than people with higher or primary education (17.3 vs. 19.7 vs. 21.5; p<0.001). No statistically significant differences was observed between the level of education and adherence in people with DM (**Table 3**).

In the comparative analysis, differences depending on the economic activity were observed only among diabetic patients. Economically inactive people in this group achieved a higher level of adherence than economically active people (16.8 vs. 17.8) (**Table 3**).

In the comparative analysis between the subjects taking into account the age difference, no correlation was observed between younger (<65 years) and older (≥65 years) people and the ARMS questionnaire result (**Tables 3** and **5**).

### Analysis of the Influence of Sociodemographic Factors on the Adherence Level: Linear Regression Analysis

An evaluation was carried out of the relationship between such variables as disease, gender, age, place of residence, marital status, education, and employment status. The analysis of the single-factor linear regression model showed the positive influence (lower score) of female gender (r=−2.44), secondary education (t=−0.569), and employment status (out of work) (t=−2.82) and the negative influence (higher score) of disease [HA (t=5.46), place of residence (countryside) (t=2.01)] on the final ARMS score. Factors that have been confirmed in the multi-factorial model are HA (t=5.35), female gender (t=−2.95), and secondary education (t=5.30) (**Table 6**).

**Table 6:** Single and multiple regression analysis of selected sociodemographic variables and their influence on the result of the ARMS questionnaire (adherence).

		ARMS - sum - linear regression									
		"Simple linear regression analysis					Multiple linear regression analysis				
		B	SE	t	p-value	ß	B	SE	t	p-value	ß
**Disease**	**Diabetes**	Ref.					Ref.				
	**Hypertension**	0.92	0.17	5.46	**<0.001**	0.14	0.99	0.19	5.35	**<0.001**	0.14
**Gender**	**Man**	Ref.					Ref.				
	**Woman**	-0.40	0.16	-2.44	**0.015**	-0.06	-0.47	0.16	-2.95	**0.003**	-0.07
**Age**		-0.01	0.01	-0.3	0.75	-0.01	–	–	–	–	–
**Address of residence**	**City**	Ref.					Ref.				
	**Countryside**	0.35	0.17	2.01	**0.044**	0.05	–	–	–	–	–
**Marital status**	**Married**	Ref.					Ref.				
	**Single**	0.03	0.18	0.17	0.87	0.00	–	–	–	–	–
**Education**	**Primary or none**	Ref.					Ref.				
	**Secondary**	-1.26	0.22	-5.69	**<0.001**	-0.16	-1.16	0.22	-5.30	**<0.001**	-0.14
	**Higher**	0.90	0.23	3.94	**<0.001**	0.11	0.39	0.25	159	0.111	0.04
**Employment status**	**In work**	Ref.					Ref.				
	**Out of work**	-0.48	0.17	-2.82	**0.005**	-0.07	–	–	–	–	–

B - unstandardized regression coefficient B; SE - standard error; t: B/standard error; ß- standardized regression coefficient ß. p<0.05 indicated a significant influence on the result of the ARMS questionnaire (adherence) by the variables. Adjusted R^2^ = 0.14. Statistically significant results were highlighted in bold (p < 0.05).

## Discussion

Determining and understanding the determinants influencing the effectiveness of chronic disease treatment is a key element of therapy planning. Specifying interventions aimed at reducing the risk of non-adherence to the treatment plan can effectively reduce the number of complications, improve the quality of treatment in the long term, and reduce the adverse effects in these patients. The source literature increasingly addresses the subject of predictors related to adherence. The most frequently discussed predictors are gender, age, education, employment status, and duration of the disease (Hyre et al., 2007; Lee et al., 2010; Jankowska-Polańska et al., 2016a; Jankowska-Polańska et al., 2016b; Jankowska-Polańska et al., 2017). The strong influence of knowledge and social support as predictors positively related to adherence has been unconditionally proven (Magrin et al., 2015). In the group of elderly patients, the issues of cognitive dysfunction and frailty as predictive factors adversely affecting the effectiveness of treatment have been additionally confirmed (Jankowska-Polańska et al., 2016a). The lack of a unified position on the sociodemographic variables and their connection to adherence has led us to seek an answer to the question whether any of the factors discussed here can be considered significant and most important in pharmacological adherence, and whether there are differences in the profile of adherence determinants depending on the chronic disease.

The results of our own study showed that patients with DM or HA have a low level of pharmacological adherence. Such a picture of patients (55%) reaches a low level of adherence, according to the results published in the literature. Adherence levels in patients with HA ranges from 10% to 92% (Grahame-Smith and Aronson et al., 2002; Carter et al., 2005; Wolf-Maier et al., 2014), and in diabetic patients the results are very similar, ranging from 38.5% to 93.1% (Krass et al., 2015**)**.

Additionally, it should be emphasized that in our study, in comparative analyses, the level of adherence by patients with HA was significantly lower than in diabetic patients, and in the analysis of regression, HA was a statistically significant independent determinant lowering adherence. The fact that patients with DM take medication more regularly may be due to the awareness that nonadherence increases risk of developing early complications of DM. The consequences of non-adherence may be early stages of hyperglycemia but also late stages, including macro- and micro-angiopathies (Quiñones et al., 2018). On the other hand, low adherence may be caused by a multitude of side effects, which may or do occur in pharmacological treatment, and which are not so common in DM. The symptoms of hypoglycemia and weight gain are most frequently mentioned in the treatment of DM, as most often related to decreased adherence (Larkin et al., 2015), whereas in the treatment of HA, headaches, edema, electrolyte disorders, coughing, hypotension, potency problems, and cardiac rhythm disorders appear (Harrison et al., 2015**)**. As can be seen from the observations of patients with HA, medications are most often taken during high BP, but once normalization is achieved, patients discontinue treatment, considering their condition as cured or not requiring medications to be taken continuously. Patients with HA may have a very liberal approach to treatment because of the lack of tangible symptoms of the disease and hardly imaginable distant consequences (Santa Helena et al., 2010; Karakurt and Kasikci, 2012). According to many researchers, the asymptomatic course of the disease and the awareness of the necessity of life-long treatment are factors that contribute to the failure to undergo treatment.

There is a discussion in the subject literature on the impact of gender on the level of adherence. A study by Jankowska-Polańska confirmed that male gender decreases the level of adherence (Jankowska-Polańska et al., 2017), but a study by Hyre et al. showed that female patients had lower adherence scores (Hyre et al., 2007). A study by Kwissa-Gajewska on 278 patients with type 2 DM who were started on insulin therapy, confirmed the correlation between the level of adherence and the gender of diabetic patients. These correlations are explained by differences in beliefs about medication, coping strategies, the level of motivation to follow treatment recommendations, and the way of communicating with medical personnel. Research shows that women cope better with treatment changes (Kwissa-Gajewska and Kroemke, 2013).

In the comparative analysis in our study, economic activity was a determinant affecting the adherence level for the entire group being studied. People who are out of work achieved a significantly higher level of adherence than those in employment, especially in the group with DM. However, economic activity was not a statistically significant independent determinant of adherence in the multiple regression analysis. It’s difficult to imagine the reasons for such a patient profile. It is highly likely that people who are out of work concentrate on the treatment process and have more time. Additionally, in people with DM, being out of work may result from the advanced age of patients, which may involve insulin therapy. The results of the available studies indicate that insulin treatment is much more frequently observed by patients as a necessity, and that the tablets can be dispensed with, which may explain the high level of adherence (Biderman et al., 2009; Bener et al., 2014).

Multiple linear regression analysis showed that among the sociodemographic determinants analyzed, female gender is important and independent predictor of adherence and improve adherence in the entire study group. It is important to note, however, that in our studies, few sociodemographic variables were equally important in HA and DM. In our own study the only determinant that was common in the comparative analysis was female gender. In both HA and DM, women achieved a better result. Studies show that women are much more interested in reporting their medical problems and use medical services (Addis and Mahalik, 2003; Santa Helena et al., 2010). The role of gender as a determinant in studies on HA patients is heavily discussed and opinions published are contradictory (Kim et al., 2003; Imtiaz et al., 2014**)**. On the other hand, in studies of diabetic patients, it has been demonstrated that there is no gender-related relationship with adherence (Tiv et al., 2012; Riaz et al., 2014; Dash et al., 2015).

Larkin’s research showed that women, elderly patients and those with type 2 DM appeared to have greater effects of education on adherence than men, younger persons, and those with Type 1 diabetes mellitus (T1DM), respectively (Larkin et al., 2015). There is no doubt that the apparent differences in the level of adherence between the genders may be due to the level of education, the resources available, health literacy, and the financial dependence of women on men (Larkin et al., 2015).

In the comparative analysis, education level was relevant only in the case of HA. The available literature indicates the important role of medical education and patient preparation for experiencing ill health and treatment. Educational programs also benefit patients with a low educational level. A review of the literature proves that the higher the level of education, the greater the benefits of medical education. It is very possible that people with higher education are more likely to participate in education feeling and understanding the need to learn, while others do not feel this to be a necessity and have no need to learn, or have difficulty in understanding and assimilating the information provided.

One more predictor influencing the level of adherence in the comparative analysis for the group of patients with HA was the place of residence. People from the city obtained a higher level of adherence than those living in the countryside. The Magnabosko study showed that the level of adherence among patients with HA was equally low in the city and in the countryside, with a slight tendency for higher adherence among patients in the city. This fact is explained by some researchers as the influence of socioeconomic and cultural aspects (Magnabosco et al., 2015). Factors not without significance include long distances from rural areas to specialist facilities and difficulties patients experience in reaching specialists (Rasella et al., 2014).

In the own study, old age and marital status were irrelevant determinants in the process of adherence. The results of our own study are not confirmed in the literature on the subject, where the role of being single as a determinant of non-adherence is very evident and the role of social support is to increase and support adherence (Świątoniowska et al., 2019). As far as age is concerned, there is still an ongoing discussion in the source literature. Authors have a different position and in part of the study, old age is conducive to adherence and in part of the results of scientific research only younger age is associated with higher adherence (Kim et al., 2003; Jankowska-Polańska et al., 2017).

The study allowed for observing similarities and differences in the profile of adherence determinants among patients with chronic DM and HA. It may be stated that due to a low level of adherence, chronic treatment, and distinctive nature of the determinants associated with treating these two most common social diseases, type 2 DM and HA require individualized treatment approach, especially including those patient groups who are predisposed to a low level of adherence, and therapeutic model adaptation to particular groups or even individuals.

## Conclusions

1. HA is an independent, statistically significant predictor that reduces the adherence level. HA patients have a lower adherence level than DM patients.
2. Female gender and higher education are the most important determinants improving adherence to pharmacological therapy.
3. There are differences between patients with HA and DM. Occupational activity plays an important role in DM, while education plays a role in HA.

### Study Limitations

Our study has several limitations. The first one is its way of measuring the level of adherence using a direct method (a self-reported questionnaire). The use of laboratory methods, such as the use of physiological markers, pharmacy records, or drug concentrations in bodily fluids for monitoring the treatment could be a supplement to the study and would verify the credibility of the self-description method. Another limitation of the study is the lack of assessment of patients’ satisfaction with the treatment, their knowledge of the disease, and beliefs about medication. The next limitation may also be that analysis was only conducted with selected sociodemographic predictors. Conducting comparative analyses on selected clinical predictors can significantly affect the final result of such analyses. One of the limitations of the study is lack of detailed age-related analyzes. In this study, it seems that “age” may be a confounding variable. Due to the very large range of age in the study groups and the categorization of this variable, which was used, it can be considered that the variable age is not adequately examined in this manuscript. The final limitation of the study is the lack of the cost of treatment and the multi-drug analyses.

## Data Availability Statement

The raw data supporting the conclusions of this article will be made available by the authors, without undue reservation, to any qualified researcher.

## Ethics Statement

The study was approved by the local Bioethics Committee (approval No 730/2019). The study was performed in accordance with the Helsinki Declaration and the principles of good clinical practice, with respect for the rights and dignity of the participants. All participants provided written informed consent.

## Author Contributions

BJ-P contributed to the conception or design of the work. JP contributed to the acquisition, analysis or interpretation of data for the work and conducted the literature review. PK, MS, and NŚ-L drafted the manuscript. EG critically revised the manuscript. All authors contributed to the article and approved the submitted version.

## Funding

This work was prepared as part of the project of NŚ and BJP, financed from funds granted by the Ministry of Science and Higher Education in the “Regional Initiative of Excellence” program for the years 2019–2022, project number 016/RID/2018/19. The funder was not involved in the study design, collection, analysis, interpretation of data, in the writing of this article, nor in the decision to submit it for publication.

## Conflict of Interest

The authors declare that the research was conducted in the absence of any commercial or financial relationships that could be construed as a potential conflict of interest.
